# Biosecurity in pig farms: a review

**DOI:** 10.1186/s40813-020-00181-z

**Published:** 2021-01-04

**Authors:** Laura Valeria Alarcón, Alberto Allepuz, Enric Mateu

**Affiliations:** 1 Facultad de Ciencias Veterinarias, Universidad Nacional de La Plata, Calle 60 y 118, La Plata, Buenos Aires Argentina; 2 Departament de Sanitat i Anatomia Animals, Facultat de Veterinària, Universitat Autònoma de Barcelona, Travessera dels Turons s/n, 08193 Cerdanyola del Vallès, Barcelona, Spain; 3 Centre de Recerca en Sanitat Animal (CreSA-IRTA-UAB), campus UAB, 08193 Cerdanyola del Vallès, Barcelona, Spain

**Keywords:** Biosecurity, Disease prevention, Mitigation measures, Pig farming, Epidemiology

## Abstract

The perception of the importance of animal health and its relationship with biosecurity has increased in recent years with the emergence and re-emergence of several diseases difficult to control. This is particularly evident in the case of pig farming as shown by the recent episodes of African swine fever or porcine epidemic diarrhoea. Moreover, a better biosecurity may help to improve productivity and may contribute to reducing the use of antibiotics. Biosecurity can be defined as the application of measures aimed to reduce the probability of the introduction (external biosecurity) and further spread of pathogens within the farm (internal biosecurity). Thus, the key idea is to avoid transmission, either between farms or within the farm. This implies knowledge of the epidemiology of the diseases to be avoided that is not always available, but since ways of transmission of pathogens are limited to a few, it is possible to implement effective actions even with some gaps in our knowledge on a given disease. For the effective design of a biosecurity program, veterinarians must know how diseases are transmitted, the risks and their importance, which mitigation measures are thought to be more effective and how to evaluate the biosecurity and its improvements. This review provides a source of information on external and internal biosecurity measures that reduce risks in swine production and the relationship between these measures and the epidemiology of the main diseases, as well as a description of some systems available for risk analysis and the assessment of biosecurity. Also, it reviews the factors affecting the successful application of a biosecurity plan in a pig farm.

## Introduction

The prevention of infectious diseases in pigs is important for both animal welfare and economic productivity. Moreover, prevention is also important for food safety and public health when zoonotic pathogens are concerned. Biosecurity embraces all aspects of the prevention of pathogens entering and spreading within a group of animals. In recent years, with the emergence and re-emergence of difficult-to-control diseases such as African swine fever or porcine epidemic diarrhoea, the perception of the critical importance of pig health and its relationship with biosecurity has increased in recent years. In other cases, for example, influenza A virus, animal pathogens have the potential for producing a pandemic event. The implementation of biosecurity measures all along the production chain minimises the risk of introduction of new pathogens into the farms, as well as their spread within farms. Nevertheless, the implementation of sustainable biosecurity programs and its continuous improvement is still a challenge for many pig farms.

### The origin of the biosecurity concept in swine production

From the decade of 1960, swine production shifted progressively from a system made of small family-owned farms towards a large-scale industry. This evolution made evident that the management of health and disease should be oriented in a new way [1]. In the 1980 decade, concepts such as “minimal disease” or “specific-pathogen free farms” began to be common and led to the modern concept of biosecurity [2, 3].

Early publications defined biosecurity as ¨the security form of transmission of infectious diseases, parasites and pests¨ [4]. However, at that time most of the available information was mainly based in a combination of knowledge on the epidemiology of some diseases, common sense and experience [4–6]. It became increasingly evident that a more methodical approach was needed. Soon thereafter scientific journals started to publish papers on biosecurity in swine farms [7–9].

### The modern concept of biosecurity

Since the early, almost intuitive, definitions of biosecurity, this concept has evolved at the same pace than swine production. Nowadays, in developed countries, pig production shows a trend towards concentration: bigger farms in lesser hands together with an increasing need of animal movements. Within this frame, the introduction of a new pathogen in a farm can have serious or even catastrophic consequences, not only for the affected farms, but also for all other connected operations. A recent example of this was the introduction and spread of porcine epidemic diarrhea virus in the Americas or of African swine fever virus in Europe and Asia [10–12]. As a result, the concept and perception of the diseases have changed from the individual to the farm and, from the farm to the region. Keeping diseases away is now one of the key elements in animal production [13].

Academically, biosecurity can be defined as the application of measures aimed to reduce the probability of introduction and spread of pathogens [5, 14]. When the measures are aimed at the reduction of the probability of introduction, the term external biosecurity is used. When the measures aim to reduce the spread of pathogens once they are already present in the farm, the term internal biosecurity is used.

The key concept in biosecurity is to avoid transmission, either between farms or within the farm. Therefore, the applicable measures must result in a reduction of the probability of effective transmission. This implies a knowledge of the epidemiology of the diseases to be avoided, particularly of the routes of transmission, the stability of the agent in the environment and the role of fomites and vectors [15].

For many important diseases, knowledge of epidemiology is much less than complete. This is an important gap that needs to be filled. However, since the ways in which a pathogen can be transmitted are limited to a few, for most diseases it is possible to foresee a set of potentially efficacious measures. A completely different point is to establish a prioritisation of the measures based on its potential efficacy. This requires a quantitative knowledge on the contribution of each route or element in the transmission of the infection. Beyond that, the actual implementation of the selected biosecurity measures involves economic, sociological and even psychological aspects.

## Main biosecurity measures

In this section we will review the most commonly applied biosecurity measures.

### Common external biosecurity measures

The concept of external biosecurity can be intuitively understood as the blocking of the farm from the “dangers coming from the outside world”. This implies that many of the measures aimed at the external biosecurity are physical barriers or rules banning the introduction of certain animals, people or vehicles.

#### Introduction of replacements, quarantines and use of semen

The highest probability of introduction of a new pathogen is the introduction of animals [5, 16–18]. Due to the nature of the current production systems, to keep productivity within the desired standards, replacement of breeders is needed. In most cases, this can imply a renewal of the whole breeding population every 2–2.5 years. Those replacements can be produced internally; namely some of the female offspring is selected as the replacement for the existing sows or, they may be purchased from an external source. Internal replacements may be convenient for some farms that operate as a closed system and rely on males (semen) for genetic improvement. Eradication of diseases endemic in the farm is often difficult when internal replacements are used. A similar reasoning applies to the use of semen produced in-farm.

In other production systems, external replacements are preferred in order to fully control all management and health aspects of the replacement gilts. In this later case, the implications of this fact are double: firstly, the higher the frequency of new entries, the higher the probability of entering a pathogen and; secondly, the higher the replacement rate, the more difficult to maintain herd immunity against the endemic farm pathogens. To this, it must be added the need for insemination doses that, if purchased from an external source, can be a risk for the introduction of new pathogens as well.

Assuming that many farms must rely on external replacement sources, the way in which those new animals are to be managed will become the key to success. At present, the most efficient way of organizing production is in mating/farrowing batches (usually every week or every 3 weeks). Ideally, this organization requires entry of replacements with the same periodicity of the farrowing batches (weekly or every 3 weeks). In these systems, one first biosecurity barrier would be to set a list of health requirements for the sources of gilts. This list must classify diseases based on the risk they pose to the farm and must indicate which verification tests are to be performed (as a routine). While for some pathogens the mere suspicion of its presence in the source farm would be enough to discard that source as a supplier (e.g., the presence of PRRSV seropositive gilts in a source aimed to supply a PRRS-negative herd), for other pathogens, their presence would be admissible under certain conditions (e.g., porcine parvovirus is acceptable since vaccination is highly effective). In any case, a well-designed and well-managed quarantine is the most effective measure to reduce risk associated to the introduction of external pathogens.

Quarantines must be designed as biocontention units; namely, they must be designed to avoid the spillover of any undesired pathogen brought by the incoming animals. Therefore, direct connection between the quarantine unit and the main farm must be blocked. Usually, this means locating the quarantine far from the main units of the farm and treating quarantines as if they still were the “outside world”; that is, managing them as independent facilities. Additionally, the quarantine has to be managed in a strict all-in/all-out system to avoid potential transmission of pathogens between different gilt batches. Risk associated to the entry of gilts can be reduced by decreasing the frequency of entry of the new batches. However, this implies changes in the whole organization of the farm and in the managing of breeding batches, with the consequent problems for allocating the animals in the available spaces. The bigger the batch, the larger the space needed for each batch.

Regarding the localisation of quarantines, most often it is said that they must be located no less than 1000 m from any other pig unit. This is considered a safe distance for airborne transmission of most pathogens (but not all), and for transmission by rodents, flies, etc. [19, 20]. However, some viral pathogens such Aujeszky’s disease virus, foot and mouth disease virus or porcine reproductive respiratory virus 2 (PRRSV2) or bacteria such as *Mycoplasma hyopneumoniae* have been reported to be transmitted -or could be potentially transmitted- by air to longer distances (up to 20 Km for foot and mouth disease virus, 9 Km for Aujeszky’s disease virus, PRRSV2 or *M. hyopneumoniae*) [21–23]. Air filtration is proven to prevent pathogen introduction in high density areas The use of HEPA air filters in the windows or ventilation inlets reduces the entrance of pathogens [24–27]. HEPA filters are the gold standard of microbiological air filtration but less expensive filters may also have a good efficiency. Under laboratory conditions, a combination of fine filters (EU class M and F) resulted in > 98% of efficiency to filter equine arteritvis virus and > 99.9% for bacteria such as *Actinobacillus pleuropneumoniae* [28]. Similarly, Dee et al. (2010) showed that MERV 14 (EU 8) filters, or multi-layered polypropylene filters treated with microbiocidal compounds were fully efficient to block PRRSV or *Mycoplasma hyopneumoniae* [27]. Other low-cost filtration methods were less efficient although provided some level of protection [25]. In an economic evaluation analysis of air filtration systems to prevent PRRSV, Alonso et al. (2013) [29] calculated a payback time between 2 and 7 years depending of the reduction of the frequency of outbreaks and on the premium received for being free of PRRSV. Provided that the quarantine is far enough from other sections of the farm, the main connection between the quarantine and the main farm will be the personnel. Transmission of pathogens from personnel to pigs is mainly related to the role of humans as fomites (clothes, boots, hair, etc.) (with the exception of some diseases such as influenza). Accordingly, barrier methods are highly effective: use of clothes, boots, gloves, etc. of exclusive use of the quarantine plus the obligation of taking a shower before leaving the quarantine are generally considered enough (section 3.1.2. below revise the general requirements for entering the farm). Use of exclusive clothes, shoes and tools together with handwashing are the minimum compulsory measures. In transmission experiments for foot-and-mouth disease those measures were able to stop transmission by caretakers of pigs but not when the used species was sheep in which case the addition of a shower was a necessary measure [30, 31] Beyond this, the potential airborne spread of pathogens from the quarantine can be minimized using a system of locked doors and windows in the pens with an adequate play of air flow and air pressures.

How long the quarantine must be prolonged? The duration of the quarantine depends on three elements: a) The incubation period of the diseases included in our “avoid” list, b) the duration of the contagious period for such diseases and, c) the time needed to establish a diagnosis [5, 32]. Accordingly, the duration will be determined by the diseases included in our list and the availability of diagnostic facilities. Moreover, animals must be inspected, preferably daily, for any sign of disease [30]. Also, it is necessary to have a contingency plan for the event of a positive result for an unwanted disease. This contingency plan may range from extending isolation of the replacement batch until the gilts are no longer a threat, to discarding just positive individuals and extending quarantine of the others with continuous monitoring, to complete depopulation of the quarantine and monitoring of the destination farm.

At this point of the review it is worth to note that quarantine and acclimation are somewhat opposed concepts. While quarantine is aimed to avoid the entry of pathogens brought by incoming animals and, therefore, minimizing contact between existing and new animals is critical; acclimation is aimed, among other goals, to develop immunity against the pathogens existing in the farm [33, 34] and, this often requires to have a close contact between newcomers and the present stock of breeders. Thus, a clear-cut separation must be made between the quarantine and acclimation phases. In Brazil, Serafini Poeta Silva et al., (2019) [35], associate the adaptation of the replacement to a lower seroprevalence and better control of Swine Influenza in breeding herds. Use of externally purchased semen is, in practical terms, equivalent to the introduction of a boar. Again, suppliers should be certified to be free of the diseases in our “unwanted” list and must be auditable with regards to their health status [18]. The number of providers should be as limited as possible (ideally, only one) to reduce the risk [36–38].

#### People and vehicles

People and vehicles can be important pathways for the introduction of new diseases in the farm [39–42]. By its own way of operation, farms receive lots of visits and vehicles: workers of the farm, veterinarians, repair workers, transports of feedstuff, dead-animals, etc. Beyond that, transport of animals is a category of its own.

Fomites carried by people (boots, clothes, etc.) or even the people itself, through contaminated skin, can spread various pig pathogens such as Salmonella, PRRSV, PED, TGEV, Brachyspira or Lawsonia [43–49]. People can also act as introducers of diseases common to people and animals. This is the case of influenza. In fact, classical H1N1 viruses or the original introduction of H3N2 originated from humans, just like the 2009 H1N1 pandemic virus (reviewed by Rajao et al.) [50].

The risk associated to visits may be minimized by a combination of barrier measures and regulations restricting the entrance to the farm. Who (people or vehicles) must be allowed to enter the farm? The generic answer to the question is simple, only those that are essential; the practical application of this principle is much more complicated. A list on who can or who cannot enter should be elaborated stating the rules for entering. In the next lines the main actions to minimize these risks will be reviewed.

The essential measure to restrict visitors and vehicles in the farm is to establish a clear delimitation of clean and dirty areas [51]. Clean areas area those inside the farm perimeter, in contact with pigs. The clean areas include barns, offices and connecting hallways and all the areas and equipment in contact with pigs Dirty areas are those that may contain sources of infection for the pigs present in the farm and, in practical terms, everything outside of the clean areas can be considered a dirty area. Entrance doors, walls, a changing room, a shower or a line painted in the floor maybe the interface between clean and dirty areas. Nothing should be allowed to cross the dirty area towards the clean area without being decontaminated. Clean and dirty roads may also exist. The dirty road must be used for any transport that serves multiple companies or farms. Work routines must be organized.

A perimeter fence with a permanently closed door that can only be opened from inside the farm is the main division between “inside” and “outside” the farm. A side use of this fence is to restrict access of wild animals such as wild boars, which are a serious risk for some diseases such as Classical or African swine fever [52–54]. To note, materials of the fence have to be chosen for that purpose, as wild boars can easily destroy regular wire fences. Beyond that, barriers preventing excavation under the fence must be built.

A parking area outside the farm must be implemented [55] for all those operations that do not require entrance to the farm with the vehicle (for example, the veterinarian visiting the farm).

Some operations require some level of contact with the farm. This is the case of vehicles delivering feedstuff, collecting dead animals or slurry. The most adequate approach for those operations is a proper design of the farm. The activities requiring such contact need to be located, as far as possible, in the external perimeter with no need to enter the farm. For feedstuff, the most adequate way is to locate the silos for feed close to the perimeter fence, allowing the loading from the truck without the need of entering the farm. Containers for dead animals and slurry tanks must be located immediately outside the perimeter fence to avoid the collecting truck entering the farm. Both vehicles circle in “dirty road”. In farms where the design does not allow that, a clear delimitation of the road and of the clean and dirty areas is essential. Under any circumstance the trucks, the drivers or other assistant personnel should be allowed to enter in contact with the animals [40, 41]. Kim et al. [48] showed the importance of this measure. They examined the transmission of porcine epidemic diarrhoea virus under low and high biosecurity measures and observed that clothes and boots of personnel exposed to infected animals easily got contaminated with amounts of virus likely causing transmission, particularly for boots and coveralls. Small amounts of contaminated faeces in the boots of a driver could be enough to infect a farm.

Once a vehicle/visitor is allowed to enter in to the farm perimeter, a set of rules to minimize risk must be applied. Entry of people must be compulsorily done through a reception building. They must register in a registry book indicating name, company and/or reason for visiting and indicating the latest day that they visited a pig farm. In many farms, safety procedures usually indicate a 24-48 h period to consider that a previous visit to a farm is not a risk. However, this is not based in a real evaluation of the lability of different swine pathogens since survival of different virus, bacteria or parasites may be very different and has not been thoroughly studied. An additional factor to be considered when establishing this period is the health status of the farm, the barriers established at entering the premises (showering, handwashing, changing clothes, etc.). In a farm with a good standard requiring at least handwashing and changing outwear and boots, the main risks would be associated to contamination of hair or to presence of pathogens in the oronasal mucosa. Kim et al. (2017) found *Porcine epidemic diarrhoea virus* RNA in the hair of personnel in contact with infected animals 1 day after contact but the positive personnel was not able to transmit the infection. Oma et al. (2018) [56] in a model of exposure to bovine viruses (bovine coronavirus and bovine respiratory coronavirus) viral RNA was not found in the oronasal mucosa of exposed people 6 h after the exposure although most of them were positive at 1 h. Taking the previous data in consideration, 24 h could be a reasonable time for a farm with a good health standard and applying basic biosecurity measures. Certainly, the higher the health standard and the potential of impact of a new disease, the longer that restriction period (up to 48 h). Introduction in the farm of laptops, cell phones and other electronics may be a risk if they are not decontaminated. Browne C., et al., (2016) [57] observed the viability of *Mycoplasma hyopneumoniae* on various surfaces for up to 8 days at 4 °C.

The next step would be to establish the rules for entering into the facilities allocating the animals. The minimum acceptable regulation would be to change clothes and boots for ones of exclusive use of the farm, washing hands and not sharing materials between farms. In those cases, that materials must be shared, it can be useful to expose them to UV irradiation [58] or to immerse them in disinfectant solution [3]. These can range from diluted bleach to commercial disinfectants. To note that most disinfectants have lower or poor activity in the presence of high concentrations of organic material. Wearing gloves, and eventually a cap, is advisable. A higher level of biosecurity would include a compulsory shower. As commented above, taking a shower and fully changing outwear is able to fully reduce transmission of FMDV between pigs or sheep by contaminated personnel [30, 31] although for many pathogens handwash and outwear change probably are effective.

In this case, again, clean and dirty areas have to be delimited. A very simple rule would be to consider any area where it is allowed to wear clothes or shoes form the “outside” as dirty. A simple separation can be a bench that delimitates the clean and dirty area of the changing room. Relevance of these measures are supported by transmission studies on Influenza, PRRS or FMD, where these viruses were transmitted through contaminated boots, gloves, contaminated skin or overalls that had been in contact with infected pigs [45, 59, 60]. Pork products consumption in the farm, either by visitors or personnel, should also be avoided as some important pathogens such as ASF can survive in them [61].

#### Transport of animals

Vehicles used to transport animals between farms or to the slaughterhouse and drivers from these vehicles can have an important role in the transmission of pathogens between farms, as it has been described elsewhere [40, 41, 62]. Several measures can be applied to reduce such risks.

The first one would be to define the uses allowed for a specific truck. A “safe” animal transport truck should not be used for a risky transport. For example, a truck destined for the transport of replacements must not be used for transport of animals to the slaughterhouse. Similarly, a truck should not pick up animals on different farms as this increases the risk of spread of pathogens. Therefore, establishing a list of “allowed transports and permissible actions” for each truck, along with the design of its routes, would be the first measure. Secondly, truck cleaning and disinfection must be done in a planned and conscientious manner. Cleaning and disinfecting trucks is a very difficult task to carry out in practice [63]. As a matter of fact, it has been shown that a high percentage of slaughterhouse trucks were positive for *Salmonella* after cleaning and disinfection procedures [63]. There is a general agreement that for this cleaning and disinfection to be effective, the process must include the removal of organic matter, cleaning with water, preferably hot and soapy or with descaling, drying and subsequent disinfection with appropriate substances [57, 58]. The main problems arise from the difficulty of removing organic residues from corners and recesses in the truck bed and from drying the trucks. In winter, particularly in cold climates, natural drying of a truck can take days. For this, alternatives such as air drying or heated boxes have been designed [64, 65]. Actually, Dee et al. (2004) [64] found that when trucks were washed, disinfected and dried, PRRS virus could not be found by RT-PCR nor transmission happened to sentinel pigs. All other methods allowed the presence of the virus.

Loading and unloading animals is one of the most critical situations regarding the contact of animals present in the farm with vehicles or persons from outside the farm. The best approach to minimize risks is to build a loading/unloading dock. This structure must have a dirty area (outside the farm) where trucks may park. This dirty area leads to a managing corridor (narrow enough to allow animals passing one by one) that has a gate. That gate should be low enough to permit only the crossing of an animal but not of a person standing. Usually, this is achieved by means of a sliding door or a similar mechanism. From the gate inwards should be considered “clean” area.

#### Neighbourhood

This term is related to the spatial clustering of cases, whereas the specific path by which transmission occurs among neighbours is not always clear [66]. For example, Torremorell et al. [67] attributed 80% of new PRRSV infections on negative commercial farms to spread from neighbours, but the exact path of transmission was not identified. The probability of infection due to the farm location will be variable and influenced by what is present in the neighbourhood [67]. The number and type of pig farms (e.g., presence of fattening farms versus breeding farms), presence of slaughterhouses, garbage dumps or dead-animals rendering plants in a radius of 1 km to the farm could increase such probability [5, 68, 69].

One possible path of pathogen transmission among neighbours is airborne spread. As mentioned before, distance by which pathogens can be transmitted through air is variable and will also be dependent on weather conditions (i.e., optimal in winter with high humidity and constant moderate winds) and on landscape (i.e., optimal in flat land).

Probably, FMDV and PRRSV have been the best studied pig pathogens with regards to airborne transmission. For FMDV it was shown that long-distance (up to 10 Km) airborne transmission was more likely to occur with high humidity (> 60%), low speed wind with stable direction, cloud cover, temperature below 27C (better at lower temperatures) and no precipitation [70–74]. For PRRSV, one of the main factors for the viability of the virus in the aerosols was temperature with a very short half-live (less than 30 min) at 20C. In the case of PRRSV, low humidity seems to favour survival in aerosols under laboratory conditions [27, 75, 76]. A 2-year study showed that cool temperatures, low sunlight levels, winds of low velocity in conjunction with gusts and rising humidity and pressure were the conditions more likely to favour PRRSV airborne transmission [27] Moreover, in the case of PRRSV2, the aerosol transmission of different strains may differ. For PRRSV1 airborne transmission seems less likely, maybe because of the lower levels of viremia [77].

The measures to prevent this transmission are basically barrier measures. The simplest is to raise a hedge or plant a grove that acts as a barrier in the most frequent direction of the wind in the area but more sophisticated systems, such as the installation of HEPA or other type of filters, can be used as mentioned before.

Other paths of pathogen transmission linked to the neighbourhood include rodents, mechanical vectors such as flies, and others animals (either stray or belonging to neighbouring farms) or birds. Rodents can be carriers of numerous pathogens that affect pigs, such as some *Salmonella* serovars, *Leptospira*, *Yersinia pseudotuberculosis*, *Toxoplasma gondii*, *Campylobacter* spp., *Brachyspira spp*, *Lawsonia intracellularis* or the encephalomyocarditis virus [78–83]. Generally, mice have a radius of action of 25 to 150 m and therefore, their role in transmission between farms is limited. However, individual rats can move 3 km away in one night [79, 84]. Flies can act as mechanical vectors, although their flight radius (2-3 km) and the narrow range of temperatures at which they survive [85] limit their role as mechanical spreaders of pathogens at large distances. Nevertheless, some studies showed the presence of infectious PRRS virus in a proportion of houseflies captured at 1.7 Km from the source farm [86]. Anyway, transmission has not been proven farther than hundreds of metres [62]. Evidences exist for the role of flies in the transmission of other pathogens such as *Streptococcus suis* or *Brachyspira spp.* [21, 87, 88].

Dogs and cats may also be the source of some pathogens for pigs [89–92] although these animals should not be present in a pig farm. A perimeter fence may prevent stray or neighbouring animals from entering into the farm premises.

Some species of birds have been associated with disease outbreaks. For example, in one study it was estimated that around 30% of new TGE outbreaks were caused by starlings [93]. Birds have also been involved in the spread of some pathogens such as *Salmonella*, *Lawsonia intracellularis*, *Brachyspira hyopdiseneteriae* and *E. coli* [83, 94–96] and may act as a reservoir perpetuating circulation on the farm. The main biosecurity measure would be the placement of bird proof nets on windows and keeping the doors closed to avoid the entry and nesting of birds. All buildings must be bird proofed. Any damage to bird netting or the facility exterior which allows pest entry must be repaired immediately. Furthermore, silos and feed tanks should be kept closed to prevent access by birds and contamination by faeces. This may be important in the case of Salmonella [70].

#### Feed and water

Feedstuff itself does not generally pose a risk due to the hygienic conditions in the production, particularly if the feed is heat-treated. For example, pelleting eliminates PEDV from contaminated feedstuff [97]. Nevertheless, different pathogens can contaminate and survive on feed ingredients and could therefore be introduced in a farm [98–103]. For example, Dee et al. (2016) detected the PEDV, ASFV, SVA, CSFV, PRV, and FMD in soybean meal (conventional and organic), vitamin D supplements, lysine and choline [99]. Actually, pigs fed with PEDV-spiked feedstuff were successfully infected, proving that this can be a potential source of spread for this virus [103]. Gordon et al. (2019) [104] reviewed the role of non-animal ingredients as a source of viral pathogens for swine.

This risk can be maintained below critical status by minimizing the likelihood that a pathogen can enter the feed supply chain, such as by excluding high-risk ingredients from facilities, extending biosecurity to mills, and considering proactive mitigation strategies [105–107]. Some of these are, develop storage facilities for incoming products ‘feed quarantine’, and determining and setting a schedule for a validated sampling method [108] of ingredients that are considered higher risk (origin animal or not animal). Limit and establish a flow of movement of people (employees in the feed mill and visitors, such as guests, truck drivers, and subcontractors people) or vehicles in or out of a facility because also has the potential to introduce contaminants into a feed manufacturing facility [109]. Several studies have shown that chemical additives for the feedstuff can be reliable methods for mitigating such risk for both viruses and bacteria [110–113]. Effective additives are organic acids such as formic, lactic or propionic, but also fatty acids and essential oils have been proven to have efficacy against certain pathogens [114]. Formaldehyde has been shown to be effective at preventing risk associated with PEDV [110, 115] as well as *Salmonella* [106, 107]. Furthermore, the use of formaldehyde in feed may lead to detrimental bacterial shifts in the pig gut [116]. Another strategy that has been proven to be effective in mitigating this risk is flushing feed manufacturing equipment with rice hulls treated with chemical compounds with antimicrobial properties such as formaldehyde or a hexanoic:octanoic:decanoic mix [117]. Therefore, feed should be provided by a reputable supplier with a recognized quality assurance system and food ingredients should not be transported in a vehicle that is used to transport pigs or other livestock [98].

Drinking water used on farms could also be a source of pathogens introduction [8]. A disease that has classically been related to water contamination is leptospirosis. *Leptospira* from rats and other animals can contaminate water, or even rats can be ingested by pigs. Furthermore, most pathogens that follow a cycle of faecal-oral transmission have the potential to be carried through the water. Silva et al., (2018) [118], develop the biosecurity vulnerability scores for PRRS the results suggest that events related transmission by air and water, and people/animals movements should be prioritized. Therefore, the bacteriological quality of the water should be checked regularly, at least once a year [119]. Water systems, tanks and pipes should be cleaned and disinfected regularly as biofilm can be a source of bacteria for pigs [32]. Also the source water treatment is an important tool in risk management. Common water treatment techniques used include physically removing chemical and biotic contaminants through filtration (reverse osmosis system and /or inactivating pathogens by applying ultraviolet light [120] or chemincal oxidant disinfectants such as chlorine, [121] cloramines and ozone.

### Common internal biosecurity measures

As previously mentioned, internal biosecurity aims to reduce the probability of the spread of pathogens once the farm has been infected. These measures can be grouped into: a) measures related to management of the herd, b) general hygiene of facilities, c) cleaning and disinfection and, d) personnel.

#### Measures related to management

The main objective of this group of measures is to control the flow of animals to avoid mixing pigs from different age groups. Usually, it is considered essential to avoid movements against the production flow. This is achieved with the strict application of an all-in / all-out system complemented by cleaning and disinfecting the facilities for the new batches of animals. This measure has been reported to be effective to reduce the circulation of pathogens [122] and to reduce the amount and variety of drug application on farms. These last authors observed that in Japanese farms where the all-in / all-out system was applied in all production stages, there was a lower use of antimicrobials for the treatment of pneumonia and oedema disease. In France, a reduction in the prevalence of *Salmonella* in pigs sent to the slaughterhouse was also observed when this measure was carried out [123].

However, this flow control is not enough for all diseases. For example, for those diseases in which transmission can occur in maternities, cross fostering, even between sows of the same batch, can contribute to the spread of the disease. This has been shown for PRRS virus, in fact, limiting adoptions is one of the measures that is usually implemented during a PRRS outbreak in maternity areas [124].

Another important fact to consider when applying management measures is that sows are the reservoir for many of the pathogens present on the farm. From the late 1970s, early weaning systems began to be studied based on the idea that certain pathogens were transmitted from mother to offspring at certain times. Separating piglets from the mother earlier would prevent this transmission and, consequently, would reduce or even eliminate the presence of certain diseases [125–127]. These techniques, while partially effective, are detrimental to pig well-being and in Europe, contravene community welfare standards. It is also important to establish a work routine that takes into account the role in disease transmission of the different age groups within the farm. The usual recommendation is to establish a workflow following the pig flow, from younger to older. Thus, personnel working in the fattening units should not enter into the nurseries after contacting fatteners or go back to a maternity from a nursery.

#### Measures related to facilities and cleaning and disinfection

The facilities should contribute to reduce the transmission of diseases or, at least, must not facilitate their spread. A very basic aspect to start with would be its design. In poorly designed or poorly planned farms it is relatively common for animals to have to move between different sections for loading, unloading or between production phases so that animals of different ages can have contact. Likewise, it is important that the facilities allow a correct organization of work and, to a certain extent, contribute to respect a separation between the different ages present on the farm. This can be achieved with physical barriers such as doors, foot baths, or intermediate areas for hand washing and changing boots.

However, all these barrier measures tend to hinder work routines. Sometimes the different areas can be painted with different colours and clothes and boots of the corresponding colour can be used to make more difficult to violate the rule of non-contact between different stages of production.

The nature of the materials used in the facilities is an important factor. The separations between pens or rooms and the floor are usually cited as the most important elements. For example, discontinuous separations between pens are known to facilitate the transmission of respiratory pathogens while solid separations facilitate the transmission of enteric pathogens [128]. On the other hand, something similar happens with floors, particularly in maternity areas. While metal and plastic floors are cleaner, they have a negative impact on comfort. Straw beds are very comfortable, but increase the risk of presentation of diarrhoea outbreaks [122]. The ventilation system should also be added to this section, since inadequate ventilation contributes to an increase in the environmental microbial load, particularly for respiratory pathogens.

Regarding hygienic measures, the most basic element is the cleaning and disinfection of the pens. Similar to what happens with trucks, pens should be cleaned first by removing organic debris, then they should be washed with soapy water, and after rinsing and drying they should be disinfected. Dione et al. [129] evaluated 276 farms in Uganda, and found a reduction in seropositivity to *Streptococcus suis* by the use of disinfectants on farms. This pathogen is rapidly eliminated by phenyl compounds, chlorine, and iodine.

The second fundamental hygienic measure refers to the administration of vaccines and drugs. Needles should be exchanged between individuals, although this is very difficult to achieve in practice. Often workers see the change of needle as a waste of time. To teach them the importance of this practice is essential. The minimum acceptable would be to use individual needles in sows and, at least, to change needles for each litter or pen.

#### Measures related to personnel

Personnel working in the farm are key elements to keep internal biosecurity. Their role is double, in one hand, they have to implement the rules and, on the other hand, they may act themselves as means for the spread of pathogens within the farm.

Personnel must know well which are the assigned areas of work and what the work routines are. For example, a worker in the fattening area should not go to a maternity hospital. Often, a colour code for walls and clothes may help to this end. This implies to have specific clothing and footwear of the corresponding colour. Obviously, this requires additional planning for cleaning and replacement and areas for changing must be designed.

Finally, measures such as the use of gloves, periodic hand washing and foot baths will lessen the impact of the worker acting as a fomite within the farm. It is known that the maintenance of foot baths requires continuous attention to avoid the excessive accumulation of organic matter. The contact time with the disinfectant required to sanitize the boot varies with the product but usually is measured in minutes. Moreover, the presence of organic materials may affect the practical efficacy or the time needed to act. In farms where is not likely that footbaths and foot bath procedures are to be followed, having specific clean boots for each area can be a good alternative.

Simply walking on a foot bath and not removing the faecal matter from the boots before entering the disinfectant solution does not reduce the number of pathogens in them [6, 130]. It is therefore recommended to first clean the boots in a preliminary foot wash, using a brush and soapy water and then, followed by the immersion of the clean boot in the disinfectant solution for at least 5 min and covering no less than 15 cm of the boot sole. This is effective for disinfection and does not waste disinfecting solution in the foot baths. Disinfecting solution must be changed preferably daily and every 3 days would be the least acceptable routine [6]. If foot baths are not an option in the farm, a less effective, but still recommended measure, could be the use of different boots for the outside and inside of the different farm buildings, with the establishment of a periodic cleaning and disinfection system for them.

Vaccines are an essential part of the internal biosecurity of animal populations. Recent advances in molecular biology make it possible to generate more effective vaccines. Many of these are used to protect production species such as pigs and/or prevent zoonosis, for example, the vaccines used to control Swine Influenza [131].

### Biosecurity assessment

When designing a biosecurity program, it may be useful to have a system allowing an objective assessment of farm biosecurity. Such assessment can be used to prioritize which biosecurity measures should be improved or implemented first in order to reduce the likelihood of disease introduction and/or spread. In addition, it might enable to monitor farm biosecurity over time and to compare it with that of other farms (benchmarking). This may be especially important when applied to an entire production system of a company. It allows planning of the production flow and to determine what contacts and risk are admissible. Therefore, biosecurity evaluations will allow to improve risk management associated with the transmission of diseases both at the farm and at the company or territory level [131]. In addition, estimates about farm biosecurity might help to calculate the benefits in production, health status or antimicrobial consumption produced by the implementation of a given measure, contributing therefore to a more precise application and to increase motivation and awareness on farmers and veterinarians [132–135].

Assessing biosecurity includes measuring the potential routes for disease transmission. The first step is to collect biosecurity practices applied on the farm. For this, epidemiological surveys including questions that evaluate the external and internal biosecurity measures applied to the different routes of pathogen introduction and spread can be used. Epidemiological connections must be investigated as well.

Several methods to assess farm biosecurity have been developed. This is presented in the next sections.

### Biosecurity assessments based on scores

The most common biosecurity assessment has been the creation of scores. Most of these scores are based on values attributed to the biosecurity practices by expert opinion panels. Some of the scoring systems evaluate measures that are common to the transmission of different types of infectious agents while others are disease-specific.

One first approach is produce a score for farm that results from summing up the scores for different biosecurity practices and setting a threshold from action [7, 136].

Researchers from Ghent University developed the Biocheck.UGent™ biosecurity scoring system [137]. In this system, biosecurity practice values, as well as the different pathways for disease transmission, are multiplied by a weight factor accounting on their relative importance, obtaining therefore a risk-based weighted score for the farm biosecurity. Sasaki et al. [138] developed a similar evaluation system named BioAsseT. Several scoring systems developed for specific pathogens (PRRS, *Brachyspira hyodysenteriae, Mycoplasma hyopneumoniae)* have been reported [139–142].

On the other hand, some authors have applied statistical methods to develop biosecurity scores based on the rank of biosecurity practices according to their importance. For example, Zang et al. [143] used a multi-criteria Decision Analysis (MCDA), a method that assesses the relative importance of biosecurity practices by pair-wise comparison of measures in order to estimate how many times more important is one measure in relation to other [144]. Silva et al. [118] applied this method to PRRS.

Silva et al. (2018) [118] used the item response theory to create a general biosecurity score in pig farms. This method is based on the notion that farms that implement some biosecurity practices will also implement others related to it. By using this method, they were able to reduce the number of variables needed to quantify the biosecurity level of pig farms, simplifying thus the method.

All of the above-mentioned systems (with the exception of the study of Silva et al. (2019) [145] have used data obtained from experts. This often introduces some bias as uses subjective estimates. Expert opinion might be influenced by different factors, mostly previous experiences, the epidemiological situation in a country, or the prevailing idea in an area, among others. However, when no sufficient data are available in the literature this is a valid option as far as some basic principles are followed [146]. An adequate selection of experts based on their knowledge, experience and background but also on the lack of conflicts of interest is paramount.

### Biosecurity assessments providing probability estimates

Multivariate statistical models [147], Bayesian Belief Networks [148] and machine-learning algorithms [149] are some of the statistical models used to quantify the probability of disease occurrence and to evaluate the impact of the implementation of biosecurity practices. Although they do not consider the biological plausibility of the included variables they may be useful methods for the development of tools for measuring, benchmarking and managing biosecurity practices as described by Silva et al. [149] for PRRSV.

Quantitative risk assessment, as described by OIE [146], may also be useful to estimate the probability of disease introduction and to prioritize biosecurity measures based on their impact on the probability of disease transmission. The ultimate goal of risk analysis is to provide evidences to support decisions taken for mitigating risk of disease spread. This type of models considers the different pathways by which a pathogen can be introduced and transmitted and, within each of them, considers the different events that should have occurred for the pathogen to be transmitted. Events may depend on others and, each of them is assigned a probability based on the best knowledge available at that time, considering uncertainty or variability. Next, the probability is determined for each pathway and globally with indication of the confidence intervals [146]. Quantitative risk assessment models have been mainly used to estimate the probability of introduction of diseases at a country level [150] and for a single disease but generic risk assessment tools are also under development [151].

Quantitative risk assessment models also have several limitations. On the one hand, they are complex and time consuming and, on the other hand, they require of many data that are not always available. Nevertheless, they have the advantage of estimating the probability of disease introduction based on existing biosecurity practices and therefore, support the decision making on which biosecurity measures should be prioritized to reduce such probability. Some attempts to use this kind of models for biosecurity assessment at farm level have been developed for other species [152] but for the moment, to the best of our knowledge, not for pig farms.

### Design and implementation of biosecurity programs

A biosecurity program can be designed for a specific disease and focus on the measures towards that disease, or it can be more generic and can be designed to reduce the risks common to different diseases. In any case, as a first step, it is advisable to establish a list of undesirable diseases and identify the routes by which they are more likely to enter the farm, so that prevention measures can be placed where they will be most effective. Biosecurity assessments described in the previous section could be useful for this task.

Once the list is set, the forms of transmission have been identified, the risks associated with each circumstance have been identified and, the measures to be applied have been selected, they must be implemented effectively. At this stage, the program development should be evaluated and followed-up leading to the modification or expansion of the existing measures. To carry out the implementation of the biosecurity program, management protocols must be generated that describe step by step the actions to be applied, together with the training of the farm staff and the professionals involved.

One of the main problems for this long-term maintenance of the program is that, if it is effective, the result will be that the entry of new diseases will not be seen or the spread of existing ones will be reduced. In other words, if the program is successful, nothing will happen and this will give a false feeling of lack of risk. This might lead to the relaxation of the implementation of biosecurity practices, which, in turn, could increase the probability of disease introduction or transmission.

The application of biosecurity in each farm is a responsibility of the industry and, ultimately, of the owners of the farms [153, 154]. However, sociological and even psychological factors must be considered. It is critical to know the attitude and the expectations regarding disease prevention of people in charge of implementing the biosecurity program [155]. Possible motivators and barriers must be considered as well.

In recent years, several studies explored the factors influencing decision-making by pig farmers, as well as their attitude towards biosecurity [156–161]. Some of the reported factors could be classified as “personal” including knowledge about the transmission of diseases and about biosecurity, gender (often women do a better implementation of biosecurity programs), age and years of experience, the personality, as well as the connection of people to sources of information (technical advice, producer network, etc.) [160–164].

Regarding the availability and credibility of information sources, different studies showed that veterinarians are the source of information in which farmers place greater confidence when animal health and biosecurity are to dealt with [162–164] but not the only one. Farmers do also consider the recommendations of other sources such as those coming from the food industry or producer groups, among others [162]. Nevertheless, increasing awareness on biosecurity and disease prevention on veterinarians have been suggested as of paramount importance to improve farm biosecurity [165].

Another factor of great impact in the application of biosecurity measures is the risk perception of a disease and its consequences on the farm. Greater application of biosecurity measures has been observed after outbreaks of diseases such as PRRS [157] or influenza [148] as well as in densely populated areas of pigs, probably due to a higher perception of the transmission risk between neighbours [160]. Producer education is also an important factor, as described by Nöremark et al. [165]. In that study, the perspectives of Swedish farmers on the incidence, control and communication related to infectious livestock diseases were investigated. Results indicated that farmers who believe that they have the necessary knowledge, have a greater sense of control and also demanded that others took responsibility for preventing the spread of disease.

Therefore, to improve the application of biosecurity measures, farmer and veterinarian’s awareness should be increased, probably using participatory methods. In this sense, various governments and institutions have developed guides, manuals and materials to persuade producers and veterinarians on why and how to apply biosecurity measures. Unfortunately, many of these manuals have little real impact as producers think that those recommendations are irrelevant or impractical, even for those who have had disease outbreaks or may receive financial support. Part of this failure is due to the low confidence on government institutions. Likewise, part of the producers believes that the responsibility for the application of the measures lies with the health organizations, particularly when the measures are intended to zoonosis control or are applied by international legal or market pressures [156, 166].

## Conclusion

Biosecurity has become an essential element of livestock production, particularly in intensive systems such as in the pig industry. The avoidance of the introduction of new pathogens and the limitation of their spread will contribute to increase the wellbeing of pigs, the productivity of the farms and will contribute to public health as well. A better knowledge of the epidemiology of the pig diseases will contribute to the design of better biosecurity programs. Moreover, the development of quantitative assessment methods will permit a more precise selection of measures and a fine evaluation of their impact. Collaboration with other branches of science such as sociology or psychology may help to the sustainable implementation of biosecurity plans.

## Data Availability

Not applicable.

## Abbreviations

PRRS: Porcine respiratory and reproductive syndrome
PRRSV: Porcine respiratory and reproductive syndrome virus
HEPA: High efficiency particulate air
TGEV: Transmissible gastroenteritis virus
FMD: Foot and mouth diseases
FMDV: Foot and mouth diseases virus
ASF: African swine fever
ASFV: African swine fever virus
PEDV: Porcine epidemic diarrhea virus
PADRAP: Production animal disease risk assessment program
MCDA: Multi-criteria Decision Analysis
OIE: World organisation for animal health
