# Correction to: Biosecurity in pig farms: a review

**DOI:** 10.1186/s40813-021-00202-5

**Published:** 2021-03-05

**Authors:** Laura Valeria Alarcón, Alberto Allepuz, Enric Mateu

**Affiliations:** 1 Facultad de Ciencias Veterinarias, Universidad Nacional de La Plata, Calle 60 y 118, La Plata, Buenos Aires Argentina; 2 Departament de Sanitat i Anatomia Animals, Facultat de Veterinària, Universitat Autònoma de Barcelona, Travessera dels Turons s/n, 08193 Cerdanyola del Vallès, Barcelona Spain; 3 Centre de Recerca en Sanitat Animal (CreSA-IRTA-UAB), Campus UAB, 08193 Cerdanyola del Vallès, Barcelona Spain

**Correction to: Porcine Health Manag 19, 348 (2021)**

**https://doi.org/10.1186/s40813-020-00181-z**

Following publication of the original article [1], we were notified of a mistake in the second author’s name.

Originally published author name: Alberto Allepuz Alberto

Corrected author name: Alberto Allepuz

The original article has been corrected.
